# Generation and Characterization of Three Novel Mouse Mutant Strains Susceptible to Audiogenic Seizures

**DOI:** 10.3390/cells13211747

**Published:** 2024-10-22

**Authors:** Elena G. Varlamova, Vera P. Kuldaeva, Natalia N. Mitina, Maria S. Gavrish, Elena V. Kondakova, Victor S. Tarabykin, Alexei A. Babaev, Egor A. Turovsky

**Affiliations:** 1 Institute of Cell Biophysics of the Russian Academy of Sciences, Federal Research Center “Pushchino Scientific Center for Biological Research of the Russian Academy of Sciences”, 142290 Pushchino, Russia; 1928lv@mail.ru; 2 Institute of Neuroscience, Lobachevsky State University of Nizhny Novgorod, 23 Gagarin Ave., 603022 Nizhny Novgorod, Russia; verunya.rubackova@mail.ru (V.P.K.); turyginanatasha@yandex.ru (N.N.M.); mary_gavrish@mail.ru (M.S.G.); elen_kondakova@list.ru (E.V.K.); tarabykinvictor@gmail.com (V.S.T.); alexisbabaev@list.ru (A.A.B.)

**Keywords:** ENU-induced mutagenesis, epileptiform activity, audiogenic epilepsy, behavioral experiments, calcium ions, signaling, receptors, gene expression, neurons

## Abstract

The mechanisms of epileptogenesis after brain injury, ischemic stroke, or brain tumors have been extensively studied. As a result, many effective antiseizure drugs have been developed. However, there are still many patients who are resistant to therapy. The molecular and genetic bases regarding such drug-resistant seizures have been poorly elucidated. In many cases, heavy seizures are instigated by brain development malformations and often caused by gene mutations. Such malformations can be demonstrated in mouse models by generating mutant strains. One of the most potent mutagens is ENU (N-ethyl-N-nitrosourea). In the present study, we describe three novel mutant strains generated by ENU-directed mutagenesis. Two of these strains present a very strong epileptic phenotype triggered by audiogenic stimuli (G9-1 and S5-1 strains). The third mouse strain is characterized by behavioral disorders and hyperexcitation of neuronal networks. We identified changes in the expression of those genes encoding neurotransmission proteins in the cerebral cortexes of these mice. It turned out that the G9-1 strain demonstrated the strongest disruptions in the expression of those genes encoding plasma membrane channels, excitatory glutamate receptors, and protein kinases. On the other hand, the number of GABAergic neurons was also affected by the mutation. All three lines are characterized by increased anxiety, excitability, and suppressed motor and orientational–exploratory activities. On the other hand, the strains with an epileptic phenotype—G9-1 and S5-1ave reduced learning ability, and the A9-2 mice line retains high learning ability.

## 1. Introduction

Epilepsy is one of the most common neurological diseases associated with a high social burden. The incidence of epilepsy in the world is 50–70 for every 100,000 people [1]. Particular attention should be paid to the development of epilepsy in children. It has been established that the incidence of epilepsy in the pediatric population is 0.5–2% [2]. It is believed that, in patients with epilepsy, neuropsychiatric disorders are heterogeneous and simultaneously affect several functions—memory and learning, executive function, emotional intelligence and constructive thinking, speech, and visual perception, which are critical aspects pertaining to childhood development [3]. Epilepsy in children leads to complex cognitive impairment (20–60% of children with epilepsy), which is five times more severe than in adults [4]. The complexity of the epilepsy prevention and treatment processes is associated with a branched cascade of epilepsy genesis, starting with genetic causes determined during the development of the embryo and ending with numerous causes associated with environmental factors (brain injuries, infections, metabolic disorders, consequences of taking medications, etc.) [5]. It is believed that 5–50% of epilepsies are post-traumatic epilepsies [6]. However, such variations in the genesis of epilepsy is associated with the complexity of this disease. Only in recent decades has it become generally accepted that epilepsy develops through a combination of a hereditary (genetic) predisposition to the disease and damaging environmental factors [7]. Research on genetic disorders is actively being carried out, including identifying gene mutations that predispose the brain to seizure activity, which may enable the use of genotyping to predict the severity of epilepsy and select an effective treatment. Most antiseizure drugs are aimed at suppressing seizure manifestations but do not affect epileptogenesis [8,9]. Some studies suggest that a preventive treatment of epilepsy may be effective when medications are administered after the first epileptic attack [1,7]. Meanwhile, other studies show that the early administration of antiseizure drugs after a brain injury does not have beneficial effects in preventing epilepsy [10]. Moreover, drug therapy may increase psychobehavioral dysfunction rather than the course of epilepsy itself [11]. The treatment of epilepsy in children is particularly challenging. A decrease in IQ has been shown to be correlated with children taking more than one antiseizure drug [12].

The cerebral cortex is responsible for higher cognitive functions, including consciousness, speech, decision making, and memory. Cerebral cortex development is a multifaceted process; disturbances at any stage of this process lead to malformations of the cortical structure and malfunctioning [13]. It is believed that about 40% of treatment-resistant epilepsies are caused by developmental cortical malformations [14]. In accordance with this, generating animal models with such malformations is useful to investigate the molecular mechanisms of some resistant forms of epilepsy. In this regard, the generation of mouse strains using ENU-induced mutagenesis is particularly promising. ENU (N-nitroso-N-ethylurea or ethylnitrosourea) is a very potent chemical mutagen that is frequently used in genetics in order to induce random point mutations in DNA. It was successfully applied in various animal models, such as mice, C.elegance, D.melanogaster, zebrafish, and others [15]. Its ability to produce single-nucleotide replacements makes it suitable to model point mutations in humans [16]. In the present study, we describe three novel mouse mutants generated by means of ENU mutagenesis. These mice demonstrate audiogenic seizures. Here, we describe the behavioral and molecular aspects of the phenotypes of these mouse strains, with the main emphasis on the regulatory networks controlling neurotransmission.

## 2. Materials and Methods

### 2.1. Generation of Mouse Mutants Using the ENU-Induced Mutagenesis Method

Experiments were approved by the Bioethics Committee of Lobachevsky State University of Nizhny Novgorod (protocol No. 82 dated 15 May 2024).

We have already described experiments for treatment with N-ethyl-N-nitrosourea or ENU (Sigma-Aldrich, No. 759-73-9) [17,18]. The animal strains obtained in this way are then used for mapping mutations and for identifying and studying possible mechanisms of the defective gene on the pathology’s development [18].

**Figure 1:**
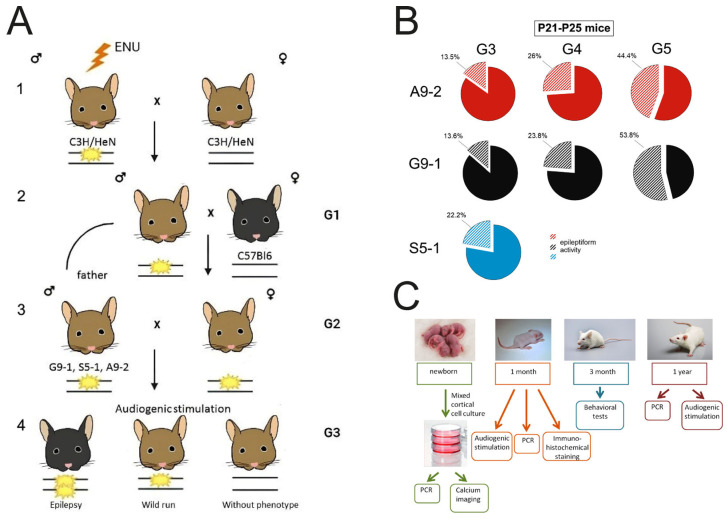
Mouse breeding scheme (**A**), the proportions of animals with epileptiform activity in offspring of different generations (**B**), and phenotyping diagram (**C**).

All mutant mouse strains presented in this study—G9-1, S5-1, and A9-2—were obtained as a result of ENU-induced mutagenesis according to the scheme presented in Figure 1.

Briefly, 8-week-old C3H/HeN mice were injected intraperitoneally with 80 μg/kg ENU mutagen. And, after a period of restoration of fertility, the injected males were crossed with intact females of the same line (C3H/HeN) to obtain the first generation (G1) of offspring (Figure 1(A1)). G1 males were then crossed with C57Bl/6 females to obtain genetic diversity (Figure 1(A2)). Subsequent mating of G2 females back to G1 fathers increased the likelihood of detecting mutations (Figure 1(A3)). At this stage, we assigned names to the generated mutant strains and used their G3 progeny to detect fixed recessive mutations by audiogenic screening (Figure 1(A4)). We then selected strains with the epileptic trait that retained it in subsequent generations. To consolidate the detected mutations in epileptic lines, various crossing methods were used, such as crossing G3 females or males with G1 individuals, backcrossing G3 males and females with G2 parents, and crossing G3 siblings with or without the mutant phenotype. The ratio of animals with epileptiform activity among offspring of the A9-2, G9-1, and S5-1 lines of different backcross generations is shown in Figure 1B. The experimental approach is shown on Figure 1C.

### 2.2. Behavioral Experiments

To compare the phenotypic characteristics of three mutant strains G9-1, S5-1, and A9-2, basic behavioral phenotyping was carried out. It included testing of motor reactions and analysis of orienting–exploratory behavior and learning ability. In addition, a specialized test was conducted for mice with a predisposition to audiogenic epilepsy. We used the following methods: measurement of the acoustic startle response, the “open field” test, counting the numbers of vertical stands, and testing the conditioned passive avoidance reflex (CPAR).

#### 2.2.1. Audiogenic Stimulation Method for Recording Epileptiform Activity in Mice

Identification and selection of species susceptible to seizures after ENU-induced mutagenesis were performed using a traditional diagnostic method for rodents—assessment of sensitivity to audiogenic stimulation [19].

The setup was based on the Startle and Fear Conditioning device, using a video camera to monitor the seizure response of mice. The previously described testing protocol, including the delivery of a sound stimulus and assessment of the convulsive reaction on the Krushinsky scale, makes it possible to accurately determine the degree of manifestation of audiogenic seizures [18]. All mice tested were age mated (21–25 days after birth).

#### 2.2.2. The Open Field Test

The tests were conducted in a square arena (45 × 45 × 45 cm) (Harvard Apparatus, Spain) with ActiTrack software (Harvard Apparatus, Spain, V 2.7.13). Animals underwent a 20 min adaptation period, after which the experimental protocol was implemented for 5 min. During the study, horizontal motor activity (HMA) was recorded at the periphery and in the center of the arena. Vertical motor activity was also assessed (based on the number of vertical stances) as an indicator of indicative and exploratory behavior.

#### 2.2.3. CPAR (Conditioned Passive Avoidance Response)

A chamber (25 × 25 × 25 cm) with an electrified lattice floor was used, divided by a partition with a hole into two identical compartments—darkened and illuminated (Panlab, Barcelona, Spain). During the training process, the mouse was placed once in the light compartment of the chamber with its back to the dark compartment. The latent period (LP) of the mouse’s stay in the light compartment of the chamber was recorded. As soon as the animal moves into the dark compartment of the chamber, it receives electrocutaneous stimulation on its paws (0.45 mA current) for 5 s. The experiment was repeated 24 h after training without presentation of an irritating stimulus, checking the integrity of the memory trace.

#### 2.2.4. Acoustic Startle Response

The experimental animal is placed in the installation in a special box for fixation. The animal’s flinching is recorded by measuring the change in the force of the animal’s pressure on the grate underneath it. Registration is carried out using computer software from Packwin (Panlab, Spain, PACKWIN 2.0.), which implements the experimental protocol. After a habituation period of 7 min, the animal is provided sound signals against a background of white noise (60 dB); the following set of signals were provided as follows:

Stimulus  (110 dB)-10 times;
Prestimul-1  (70 dB)-10 times;
Prestimul-2  (80 dB)-10 times;
Prestimul-3  (85 dB)-10 times;
Prestimul-4  (90 dB)-10 times;
No sound  stimulus-10 times.

Sequential presentation of the prestimulus and stimulus is carried out with an interval of 100 ms, 10 times with each prestimulus.

### 2.3. Preparation of Brain Slices

Fixation of mouse brain samples was carried out on the 21st day of postnatal development, after audiogenic testing. For fixation of samples and their histological analysis, the method of transcardial perfusion was used under general isoflurane anesthesia (3.0–4.0% isoflurane mixed with oxygen for 1–2 min to induce anesthesia; 1.5–2.0% mixed with oxygen to maintain anesthesia). This ensures the delivery of a chemical fixative (44% paraformaldehyde in phosphate buffer (PFA in PBS) to the brain tissue to preserve structures and morphology. The pre-anesthetized mouse was secured on a special table. The soft tissues in the sternum area and the diaphragm were dissected to provide access to the heart. During this procedure, phosphate buffer (PBS) was first passed through the systemic circulation under pressure, then a 4% paraformaldehyde solution (PFA), after which the brain was removed and immersed for a day in a PFA solution for additional fixation. The PFA in Eppendorf was then replaced by 15% followed by a 30% cryoprotectant solution (sucrose solution in 1× PBS). Incubation in these solutions was also carried out for 24 h, after which the brain was frozen (−25 °C) and cryotomy of the samples was performed. Frontal cryosections of the brain 50 μm thick were obtained. Microtomy was performed on a Leica CM1520 cryostat (LeicaBiosystems, Wetzlar, Germany) with an electromechanical knife feed system. Cryotomy was performed with the cryotome blade tilted at 10° and the chamber temperature at −20 °C. Finished sections are immersed in 1× PBS for storage (at +4 °C) and subsequent immunohistochemical staining.

### 2.4. Immunohistochemical Staining of Brain Slices

Immunohistochemical studies were performed on brain samples from A9-2, S5-1, and G9-1 mice at P21. The brains were fixed by intracardiac perfusion with 4% PFA/PBS (PanReac AppliChem, Barcelona, Spain) and then sequentially incubated in 15 and 30% sucrose. Coronal cryosections with a thickness of 50 µm were prepared using a Leica TCS SP5 cryotome (Leica, Wetzlar, Germany). The brain slices were stained with antibodies against gamma-aminobutyric acid (Rabbit Anti-gaba 1:300, Cat. #A2052, Sigma Aldrich, USA) to visualize GABAergic neurons [18]. The slices were incubated for 30–60 min in a blocking solution: 10% FBS (Fetal Bovine Serum, Cat. # A5670401, Thermo Fisher Scientific, Waltham, MA, USA), 5% triton X-100 (MP Biomedicals, Irvine, CA, USA) in 85% 1× PBS. Then, overnight at +4 °C, the slices were incubated with primary rabbit antibodies against GABA 1:300. After overnight incubation, the slices were washed three times in PBS (1× PBS in MQ water, Cat. # sc-24947, ChemCruz, Santa Cruz, CA, USA) and the histological marker was updated and incubated for 4 h with Goat Anti-Rabbit IgG 1:500 (Cat. #ab6939 Rockland Immunochemicals, Philadelphia, PA, USA), diluted in a blocking solution. After 4 h incubation, the slices were washed in PBS, placed on glass slides, allowed to dry, and mounted with mounting medium (SP15-100, Cat. #181129, Fisher chemical, Pittsburgh, PA, USA) under coverslips.

To visualize the cells in the slices, a laser scanning microscope LSM 800 (CarlZeiss, Oberkoche, Germany) was used. An argon laser was used to excite fluorescence of secondary antibodies. A series of z-plane fluorescence images were acquired and combined into a 3D stack to reconstruct a 3D image of the slice area. Image analysis was performed using ImageJ (developed by LOCI at the University of Wisconsin, Madison, WI, USA, available at https://imagej.nih.gov/ij/download.html, accessed on 18 May 2023, RRID: SCR_003070) and the Cell Counter plugin, which enables counting GABA^+^-positive neurons. Statistical analysis of the results was carried out using Graph Pad Prism (GraphPad Software, RRID: SCR_002798, V 8.0.1). To determine the normality of the distribution of the obtained values, the Shapiro–Wilk test was used. With a normal distribution of data, we used Student’s *t*-test for pairwise comparison of samples. When *p* < 0.05, the results were considered statistically significant.

### 2.5. Cortical Culture Preparation

Mixed neuroglial cell cultures were prepared as described in detail previously [20]. The cortex of one mouse was used to obtain ten Petri dishes with culture to avoid the variations in the gene expression and signaling system activity between individual mice. Briefly, 0–1-day-old pups were euthanized by halothane overdose and decapitated. The mouse cerebellar cortex was excised with clippers, put in a test-tube, incubated for 2 min, and the supernatant was removed with a pipette. The cells were then covered with 2 mL trypsin (0.1% in Ca^2+^- and Mg^2+^-free Versene solution, Cat. #59427C, SAFC, Taufkirchen, Germany) and incubated for 10 min at 37 °C under constant shaking at 600 rpm. Trypsin was then inactivated by equal volume of cold embryo serum, and the preparation was centrifuged at 300× *g* for 5 min. The supernatant was discarded and cells were washed twice with Neurobasal A medium (Cat. #10888022, Thermo Fisher Scientific, Waltham, MA, USA) before being resuspended in Neurobasal-A medium containing glutamine (0.5 mM, Cat. #G7513, Sigma-Aldrich, Burlington, MA, USA), B-27 (2%, RRID: CVCL_A315, Thermo Fisher Scientific, Waltham, MA, USA) and gentamicin (20 μg/mL, Cat. #G1397, Sigma-Aldrich, Burlington, MA, USA). Further, 200 μL of the suspension was put in a glass ring (internal diameter of 6 mm) resting on a round 25 mm coverslip (VWR International, Cat. #48382-085, Radnor, PA, USA), which has been coated with poly-L-lysine. The glass ring was removed after a 5 h incubation period in a CO_2_ incubator (37 °C), and culture medium (2/3 of the volume) was replaced every 3 days. For the experiments, cell cultures were used on days 10 and 14 of in vitro cultivation (DIV).

### 2.6. Fluorescent Ca^2+^ Measurements

Experiments were carried out in the daytime. The measurements of [Ca^2+^]_i_ were performed by fluorescence microscopy using Fura-2/AM (Cat. #F1221, Thermo Fisher Scientific, Waltham, MA, USA), a ratiometric fluorescence calcium indicator. Neurons were loaded with the probe dissolved in Hanks balanced salt solution (HBSS) composed of (mM): 156 NaCl, 3 KCl, 2MgSO_4_, 1.25 KH_2_PO_4_, 2 CaCl_2_, 10 glucose, and 10 HEPES, pH 7.4, at a final concentration of 5 μM at 37 °C for 40 min with subsequent 15 min washout [20]. Coverslip containing the cells loaded with Fura-2 was then mounted in the experimental chamber. To measure free cytosolic Ca^2+^ concentration, we used the Carl Zeiss Cell Observer and an inverted motorized microscope Axiovert 200M with a high-speed monochrome CCD camera AxioCam HSm with a high-speed light filter replacing system, Ludl MAC5000. Fura-2 excitation and registration were recorded using a 21HE filter set (Carl Zeiss, Oberkoche, Germany) with excitation filters BP340/30 and BP387/15, beam splitter FT-409 and emission filter BP510/90, objective lens Plan-Neo fluar 10×/0.3, and excitation light source HBO 103W/2. Calcium responses were shown as a ratio of fluorescence intensities of Fura-2 excitation at 340 and 380 nm [20,21,22]. ImageJ 2002 software (RRID: SCR_003070) was used to analyze data. 

### 2.7. Extraction of RNA

MagMAX mirVana Total RNA Isolation Kit (Thermo Fisher Scientific, Cat. #A27828) was used for the extraction of total RNA. The RNA quality was estimated by electrophoresis in the presence of 1 μg/mL ethidium bromide (2% agarose gel in Tris/Borate/EDTA buffer). The concentration of the extracted RNA was determined with NanoDrop 1000c spectrophotometer. RevertAid H Minus First Strand cDNA Synthesis Kit (Cat. #K1631, Thermo Fisher Scientific, Waltham, MA, USA) was used for reverse transcription of total RNA.

### 2.8. Real-Time Polymerase Chain Reaction (RT-qPCR)

Each PCR was performed in a 25 μL mixture composed of 5 μL of qPCRmix-HS SYBR (Evrogen, Moscow, Russia, Cat. #PK147L), 1 μL (0.2 μM) of the primer solution, 18 μL water (RNase-free), and 1 μL cDNA. DTlite Real-Time PCR System (DNA-technology, Moscow, Russia, 2017) was used for amplification. Amplification process consisted of the initial 5 min denaturation at 95 °C, 40 cycles of 30 s denaturation at 95 °C, 20 s annealing at 60–62 °C, and 20 s extension step at 72 °C. The final extension was performed for 10 min at 72 °C. All the sequences were designed based on the analysis of the nucleotide sequences of the existing gene isoforms and are specific to the mice with FAST PCR 5.4 and NCBI Primer-BLAST software. The sequences of all primers used in real-time PCR are presented in Table 1. The data were analyzed with DTlite software (DNA-technology, V7.9 (5.46 27 October 2022)), Moscow, Russia, 2017) and Origin 8.5 software (OriginLab Corporation, Northampton, MA, USA, SR1 b161). The expression of the studied genes was normalized to gene encoding Glyceraldehyde 3-phosphate dehydrogenase (GAPDH) and was presented regarding WT mice). The sequences of all primers used in RT-PCR are given in Table 1. Data were analyzed using Livak’s method.

### 2.9. Statistical Analysis

All values are provided as mean ± standard error (SEM) or as individual Ca^2+^ signals. All presented data were obtained from at least three replicas from cell cultures from 2–3 different cell isolations (mice). Statistical analyses were performed by paired *t*-test. n/s—data not significant (*p* > 0.05), *** *p* < 0.001, ** *p* < 0.01, and * *p* < 0.05. MS Excel (Microsoft Office 2016, Redmond, Washington, DC, USA), ImageJ (https://imagej.nih.gov/ij/download.html (accessed on 18 May 2023), Java 1.6.0_12, RRID: SCR_003070, LOCI, University of Wisconsin, Madison, WI, USA), Origin 2016 (OriginLab, Northampton, MA, USA), and Prism GraphPad 7 (GraphPad Software, RRID: SCR_002798) software were used for data and statistical analysis.

## 3. Results

### 3.1. Behavioral Analysis of Three Mouse Strains

The generation of mutants susceptible to audiogenic seizures was described elsewhere [16]. Here, we describe three other mutant strains generated from the same screening. These strains were designated G9-1, A9-2, and S5-1.

In order to prove whether the phenotypes observed were due to monogenic mutations, mice whose offspring showed the audiogenic seizures were backcrossed to C57Bl6 wild type mice for several generations. Siblings from the same generation were intercrossed or crossed with their parents. The frequencies of seizures among the littermates were between 13% and 53%. High numbers of seizures within a litter were observed when one of the parents was susceptible to seizures (Figure 1). Based on these data, we concluded that all three mutations were recessive and each mutation affected one gene.

The main goal of this work was to characterize the phenotypes of these three mutant strains. The experiments aiming to map mutations to certain genetic loci have not been performed at this stage. 

As a part of phenotyping the above-mentioned mouse strains, we characterized their behavior. In the experiments described below, we grouped all the mice that demonstrated audiogenic seizures and compared them with their littermates that did not present them. The former were designated “mutants” and the latter “WT” mice, although we assume that many mice that did not show the phenotype were heterozygous on mutant alleles. 

**Figure 2:**
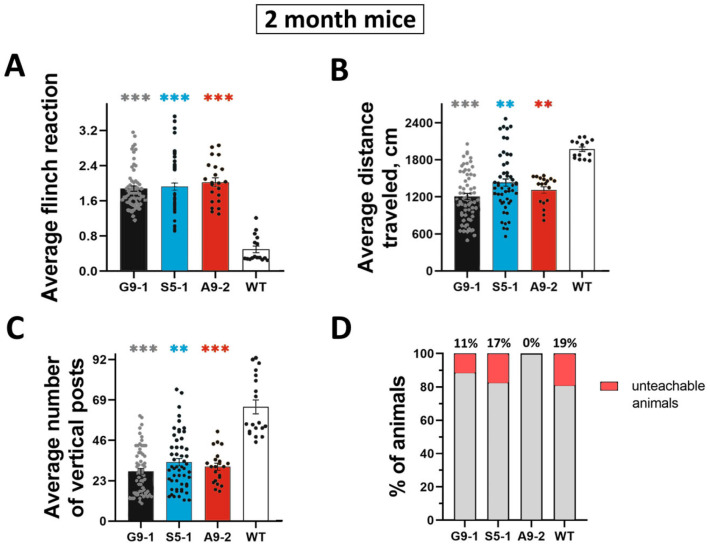
Representative indicators of basic behavioral phenotyping for 3 mutant mice lines for the acoustic startle response (**A**), for general motor activity in the mouse open field test (**B**), when assessing orienting–exploratory activity (**C**), and learning ability in the CPAR test (**D**). Data are presented as mean ± SD. The number of mice used for the experiments presented in panel (**A**) was 67 mice for G9-1 strain, 39 for S5-1 strain, 22 for A9-2 strain, and 25 for WT. The number of mice used for the experiments presented in panel (**B**) was 60 mice for G9-1 strain, 55 for S5-1 strain, 18 for A9-2 strain, and 13 for WT. The number of mice used for the experiments presented in panel (**C**) was 71 mice for G9-1 strain, 52 for S5-1 strain, 22 for A9-2 strain, and 19 for WT. ***, **—significance between the “S5-1, G9-1, A9-2” groups compared to the “WT” group (***—*p* < 0.001, **—*p* < 0.01, normality criterion Kolmogorov–Smirnov, Mann–Whitney test, *t*-test).

The mice of the G9-1 line had high rates of acoustic startle response (1.88 ± 0.06) relative to the WT group (0.62 ± 0.16) (Figure 2A, gray and white bars). In the open field test, the mice of this line showed reduced motor activity (1204.65 ± 50.85) relative to the WT mice (1874.65 ± 79.18), while their orienting–exploratory activity also turned out to be significantly lower (28.32 ± 1.70) than in the WT individuals (55.31 ± 6.41) (Figure 2B,C, black and white bars). When assessing the cognitive functions, it was revealed that only 11% of the animals from experimental group G9-1 did not develop a conditioned passive avoidance reflex (Figure 2D, column G9-1).

The S5-1 mice showed increased excitability (1.96 ± 0.09) when testing the acoustic startle response compared to the WT group (0.62 ± 0.16) (Figure 2A, blue and white bars). The average motor activity of the mice of this mutant under open field test conditions was significantly lower (1433.97 ± 62.04) than the WT animals (1874.65 ± 79.18) (Figure 2B, blue and white columns). These mice demonstrated lower levels of anxiety estimated by a higher number of vertical stances (33.57 ± 2.16) (Figure 2C, blue column). When checking the integrity of the memory trace, 17% of the mice from the S5-1 mutant line did not develop the conditioned passive avoidance reflex (Figure 2D, column S5-1).

The A9-2 mice showed increased excitability when testing the acoustic startle response (2.09 ± 0.13) compared to the WT group (0.62 ± 0.16) (Figure 2A, red and white bars). The motor activity in this line was moderately reduced, as evidenced by the open field test data: the average motor activity (1504.25 ± 109.58) is less than in those mice without the phenotype (1874.65 ± 79.18) (Figure 2B, red and white columns). The average orienting–exploratory activity of the A9-2 line turned out to be significantly lower (32.82 ± 2.75) compared to the WT mice (33.57 ± 2.16). This may indicate a decrease in basic activity as a result of a state of anxiety (Figure 2C, orange and white columns). The mice of this line demonstrated high performance in the experiments on the development of a conditioned reflex: 100% of the animals developed a conditioned reflex of passive avoidance (Figure 2D, column A9-2). At the same time, 19% of the wild type of mice were unteachable (Figure 2D).

Thus, all three mutant strains are characterized by increased excitability with reduced rates of motor and orientation–exploratory activity compared to the wild-type mice. On the other hand, both the G9-1 and S5-1 mice have low learning ability, while the learning ability of the A9-2 mice is higher than that of the WT mice.

**Figure 3:**
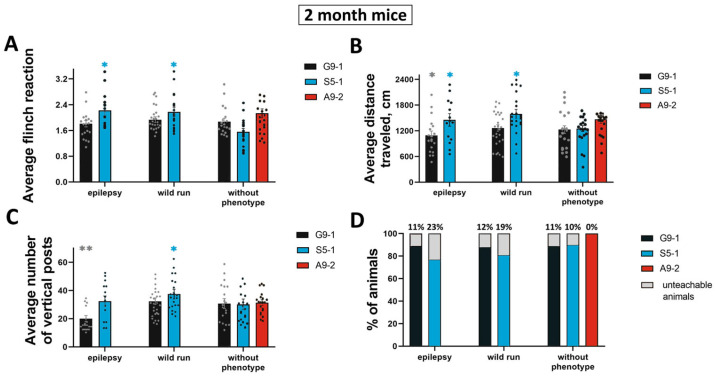
Representative indicators of basic behavioral phenotyping for 3 mutant mice for the acoustic startle response (**A**), for general motor activity in the mouse open field test (**B**), when assessing orienting–exploratory activity (**C**), and learning ability in the CPAR test (**D**). For experiments, the number of experimental mice in the “epilepsy” group was 18 mice from G9-1 strain and 10 ± 3 mice from S5-1 strain; in the “wild run” group, 26 ± 2 mice from G9-1 strain and 21 mice from S5-1 strain; and in the “without phenotype” group, 20 mice from G9-1 strain, 16 ± 4 mice from S5-1 strain, and 18 ± 3 mice from A9-2 strain were studied. Data are presented as mean ± SD. *, **—significance between the groups “S5-1, G9-1, A9-2” compared to the group “WT” (*—*p* < 0.05, **—*p* < 0.01 Kolmogorov–Smirnov normality test and test Mann–Whitney, *t*-test).

Different species within every mutant strain had varying degrees of seizure activity: motor excitation (designated “wild run”), epileptic seizures (designated “epilepsy”), and animals without an epileptic phenotype detectable in vivo. We compared the phenotypic parameters of the three animal groups: the mice of lines G9-1, S5-1, and A9-2 divided according to the audiogenic response scale. In those rodents with a predisposition to audiogenic epilepsy, the structure of the inner ear and hearing acuity are often impaired; the flinching of inbred strains insensitive to sound in response to the sequential presentation of a series of prestimuli and stimuli will differ from the reaction of individuals prone to audiogenic seizures [23]. When testing the acoustic startle reaction, the individuals of the S5-1 line with the epilepsy phenotype showed increased excitability (2.22 ± 0.16) compared to the G9-1 mice line (1.81 ± 0.09), which correlates with a reduced acoustic response in the individuals from the “no phenotype” group of the S5-1 line. In the “wild run” group, the S5-1 mice also showed high rates of the acoustic startle response (2.17 ± 0.15) compared to the G9-1 line (1.93 ± 0.09) (Figure 3A, blue column).

To test the motor and orientation–exploratory activity, the mouse open field setup was utilized. The motor activity of the S5-1 mutant mice was increased, as indicated by the data from testing in the open field; the indicators of average motor activity in the mice with the “epilepsy” phenotype (1455.08 ± 145.64) and wild run (1592.26 ± 106.59) were higher relative to the mice of the other lines (Figure 3B, blue column). The average orienting and exploratory activity in terms of the number of vertical stances of the S5-1 mice with the “wild run” phenotype turned out to be higher (37.48 ± 3.51) compared to the mice of this phenotype from the G9-1 line (32.39 ± 2.45) (Figure 3C). Also, in the G9-1 line with the “epilepsy” phenotype, a decrease in basic motor activity was noted (1095.37 ± 99.20 for the “epilepsy” group; 1263.20 ± 79.36 for the “wild run” group (Figure 3B, black column). Probably, such indicators were recorded in the mice in a state of anxiety, as evidenced by the reduced number of vertical stances (20.01 ± 2.20 for the “epilepsy” group; 32.38 ± 2.45 for the “wild run” group) relative to the indicators of the S5-1 line (Figure 3B, black column).

We also tested the preservation of the memory trace in mice from different phenotypic groups using the CPAR reaction. The conditioned reaction of passive avoidance is based on the innate mink reflex of rodents—the preference for a limited darkened space [24]. According to the test results, it was revealed that the animals from the A9-2 experimental group without an epileptic phenotype have the best learning ability since 100% of the animals developed a conditioned passive avoidance reflex (Figure 3D, red column). The S5-1 mice line with the “epilepsy” phenotype showed the worst learning ability: in the “epilepsy” group, 23% of the animals did not develop a conditioned reflex, and, in the “wild run” group, it was 19% (Figure 3D, blue columns). The G9-1 mice were marked by almost identical rates of conditioned response for mice with the phenotype—10 and 11%—and animals without the mutant phenotype—11% (Figure 3D, black bars). 

It should be noted that the A9-2 mice of the generations after G4 did not manifest strong signs of seizures such as “wild run” and “epilepsy” upon audiogenic stimulation. We concluded that such aspects of the epilepsy phenotype were genetic-background-dependent. Indeed, the contribution of the C57B6 genetic background is greater in the late backcrosses than that of the C3H background. On the other hand, the A9-2 mice demonstrated strong differences in the aspects of the phenotype described below. 

The behavioral tests carried out with the G9-1, S5-1, and A9-2 mouse lines demonstrated similarity in behavior. The following features were identified: in the mice of the S5-1 and G9-1 lines, increased excitability remained in the acoustic startle response test. Various indicators of general motor and orienting–exploratory activity fluctuated depending on the degree of the convulsive reaction observed before the behavioral tests. 

### 3.2. Number of GABAergic Neurons in the Somatosensory Cortex Is Affected in the Mutant Mice

**Figure 4:**
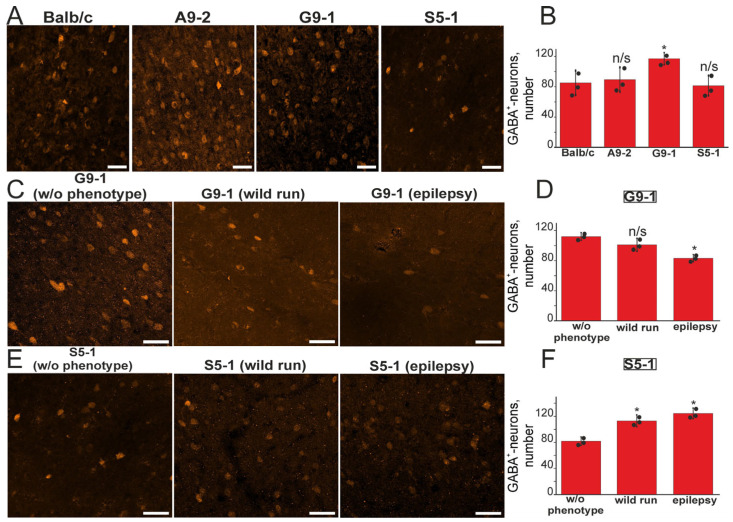
Immunohistochemical staining of the somatosensory cortex of control (Balb/c), A9-2, G9-1, and S5-1 mice with anti-GABA antibodies. (**A**) Images of brain slices of the somatosensory cortexes of Balb/c mice and A9-2, G9-1, and S5-1 mutants without an epileptic phenotype, stained with antibodies against GABA. (**B**) Average number of GABAergic neurons in the somatosensory cortexes of Balb/c, A9-2, G9-1, and S5-1 mice. For panel (**B**), statistical comparisons were conducted relative to the Balb/c group. n/s—data not significant (*p* > 0.05), * *p* < 0.05. (**C**,**E**) Immunohistochemical staining of the somatosensory cortexes of G9-1 (**C**) and S5-1 (**E**) mice after experiments on audiogenic stimulation of epileptiform activity with antibodies against GABA. (**D**,**F**) Average number of GABAergic neurons in the somatosensory cortexes of G9-1 (**D**) and S5-1 (**F**) mice. For panels (**D**,**F**), statistical comparisons were conducted relative to the w/o phenotype group. n/s—data not significant (*p* > 0.05), * *p* < 0.05. The results of immunohistochemical staining of slices from 3 mice for each experimental group were used. Scale bar—50 μm.

Next, we tested the hypothesis that the change in the number of inhibitory GABAergic interneurons can cause epileptic seizures. In order to test this hypothesis, we used immunohistochemistry with an antibody against GABA (Figure 4A). In the initial experiments, we compared the number of GABA neurons between the three lines and wild type animals without separating them into different phenotypes. The G9-1 mice demonstrated a significant increase in the number of GABA-positive interneurons in the cerebral cortex (Figure 4B). In contrast, no differences have been detected in the average number of GABA^+^ neurons in the cortexes of the S5-1 and A9-2 strains compared to the wild type mice (Figure 4B).

Next, we compared the numbers of GABA neurons within every line between animals with different phenotypes. Since the A9.2 mice did not exhibit an epileptic phenotype upon audiogenic stimulation in the late generations, a histological analysis was carried out for the G9-1 and S5-1 mice only. After audiogenic stimulation and phenotype onset detection, the mice were anesthetized, sacrificed, and the number of GABAergic neurons in the somatosensory cortex was quantified. Figure 4C shows the images of the brain slices of the G9-1 littermate’s mice without the phenotype (w/o phenotype) with epileptic audiogenic seizures, “wild run” and wild type mice. In the G9-1 mice with the “epilepsy” phenotype, the number of GABA^+^ interneurons was significantly lower compared to the other experimental groups (Figure 4D). Staining of the somatosensory cortical slices from the S5-1 mutants (Figure 4E) with antibodies against GABA showed that the groups of “wild run” and “epilepsy” contained a significantly larger number of GABA^+^ interneurons compared to those mice without the phenotype (Figure 4F).

Thus, the histochemical analysis of the GABA^+^ interneurons number in the somatosensory cortexes of the mice without the “epilepsy” phenotype showed that, in the G9-1 mice, the number of GABA^+^ interneurons is higher compared to the control mice or other mutants. In the A9-2 mice, no changes in the number of GABA^+^ interneurons were detected. Our results indicate that, in both the G9-1 and S5-1 mutants, there is an abnormal excitation–inhibition balance due to the change in the number of GABA^+^ interneurons. This in turn is likely to be the main reason for the high susceptibility of these strains to audiogenic seizures. On the other hand, while the G9-1 mice are characterized by a decrease in the number of GABAergic neurons, in the S5-1 mice, on the contrary, the number of GABA^+^ interneurons increased.

### 3.3. ENU-Induced Mutations Cause Changes in the Expression of Genes Encoding Membrane Ion Channels, Protein Kinases, GABA, and Glutamate Receptors

**Figure 5:**
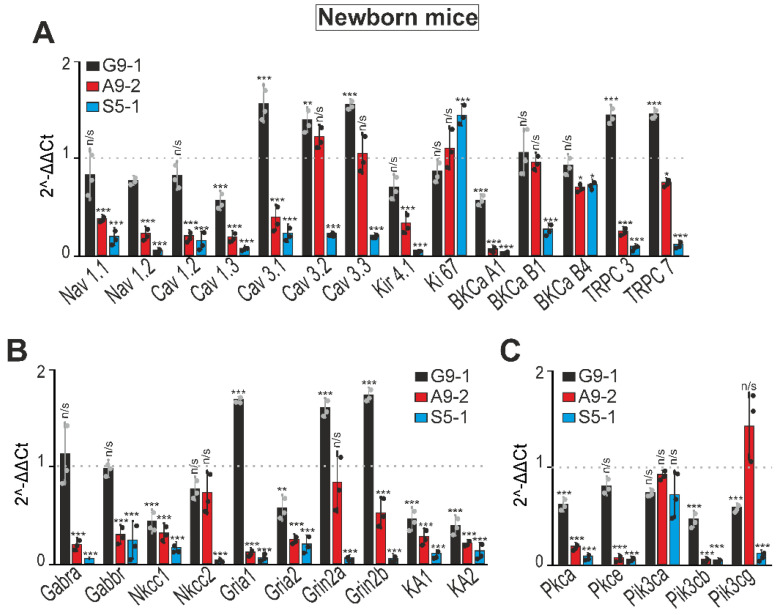
Expression patterns of genes encoding membrane ion channels (**A**), GABA and glutamate receptors (**B**), and protein kinases (**C**) in the cerebral cortexes of newborn G9-1, A9-2, and S5-1 mice. 1 is the dotted line; the level of expression in the cerebral cortexes of WT mice is taken. Results are presented as mean ± SEM. Total RNA was obtained from cortex tissue of newborn mice. Black asterisks indicate the differences between the experimental groups comparable to the WT mice group. n/s—data not significant (*p* > 0.05), *** *p* < 0.001, ** *p* < 0.01 and * *p* < 0.05. Abbreviations: Nav1.1 (SCN1A)—sodium channel protein type 1 subunit alpha; Nav1.2 (*SCN2A)*—sodium channel protein type 2 subunit alpha; Cav1.2 (CACNA1C)—calcium voltage-gated channel subunit alpha1 C; Cav1.3 (CACNA1D)—calcium voltage-gated channel subunit alpha 1D subunit; Cav3.1 (CACNA1G)—calcium voltage-gated channel subunit alpha1 G; Cav3.2 (CACNA1H)—calcium voltage-gated channel subunit alpha1 H; Cav3.3 (CACNA1I)—calcium voltage-gated channel subunit alpha1 I; Kir4.1 (KCNJ10)—potassium inwardly rectifying channel subfamily J member 10; Ki67 (MKI67)—marker of proliferation Ki-67; BKCa A1 (KCNMA1)—potassium calcium-activated channel subfamily M alpha 1; BKCa B1 (KCNAB1)—potassium voltage-gated channel subfamily A regulatory beta subunit 1; BKCa B4 (Kcnma1)—potassium calcium-activated channel subfamily M alpha 1; TRPC3— transient receptor potential cation channel subfamily C member 3; TRPC7—transient receptor potential cation channel subfamily C member 7; Gabra (GABRA1)—gamma-aminobutyric acid type A receptor subunit alpha1; Gabbr (GABBR2)—gamma-aminobutyric acid type B receptor subunit 2; Nkcc1 (*SLC12A1)*—Na–K–Cl cotransporter isoform 1; Nkcc2 (*SLC12A2)*—Na–K–Cl cotransporter isoform 2; Gria1—glutamate ionotropic receptor AMPA type subunit 1; Gria2—glutamate ionotropic receptor AMPA type subunit 2; Grin2a—glutamate ionotropic receptor NMDA type subunit 2A; Grin2b—glutamate ionotropic receptor NMDA type subunit 2B; KA1 (GRIK4)- glutamate ionotropic receptor kainate type subunit 4; KA2 (GRIK5)—glutamate ionotropic receptor kainate type subunit 5; Pkca (PRKCA)—protein kinase C alpha; Pkce (PRKCE)—protein kinase C epsilon; Pik3ca (PIK3CA)—phosphatidylinositol-4,5-bisphosphate 3-kinase catalytic subunit alpha; Pik3cb (PIK3CB)—phosphatidylinositol-4,5-bisphosphate 3-kinase catalytic subunit beta; Pik3cg (PIK3CG)—phosphatidylinositol-4,5-bisphosphate 3-kinase catalytic subunit gamma.

We hypothesized that another reason for epileptic seizures could be improper excitation/inhibition balance due to changes in the expression of the genes encoding the proteins important for neurotransmission. In order to test this, the total RNA from the cerebral cortexes of the mutant mice was isolated and the expression levels of the genes encoding the ion channels, key protein kinases, and ionotropic receptors were assessed (Figure 5). In the cerebral cortexes of the newborn G9-1 mice, we detected increased expression levels of the Cav 3.1., Cav 3.2, Cav 3.3, TRPC3, and TRPC7 genes (Figure 5A, black bars), whereas, in the A9-2 (Figure 5A, red columns) and S5-1 (Figure 5A, blue columns) mutants, the expression of almost all these genes was suppressed. In the cortexes of the G9-1 mutants, there was also an increase in the expression of the Gria1, Grin2a, and Grin2b genes (Figure 5B, black bars), while, in the A9-2 mice (Figure 5B, red columns) and S5-1 mice (Figure 5B, blue bars), the expression of these genes decreased or showed a moderate downregulation trend. In all three mutant mouse strains, the expression of the KA1 and KA2 genes was reduced in the cerebral cortex, with the most pronounced reduction observed in the S5-1 mice (Figure 5B). The expression of the Gabra and Gabbr genes in the cortexes of newborn G9-1 mice was at the level of the WT mice, while in the A9-2 and S5-1 mutants the level of these genes decreased (Figure 5B). The expression of the Pkca and Pik3cb genes encoding protein kinases C and A, respectively, decreased in all three strains (Figure 5C). In the S5-1 mice, the expression of the Pkce and Pik3cg genes was also suppressed (Figure 5C, blue bars).

**Figure 6:**
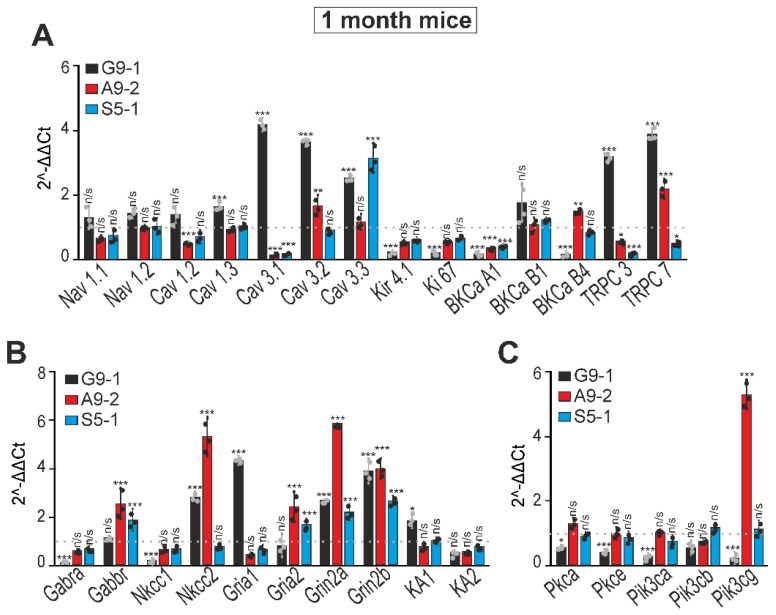
Expression patterns of genes encoding membrane ion channels (**A**), GABA and glutamate receptors (**B**), and protein kinases (**C**) in the cerebral cortexes of G9-1, A9-2, and S5-1 mice at the age of 1 month. 1 is the dotted line; the level of expression in the cerebral cortexes of WT mice is taken. Total RNA was obtained from cortex tissue of 1-month-old mice. Results are presented as mean ± SEM. Black asterisks indicate the differences between the experimental groups comparable to the WT mice group. n/s—data not significant (*p* > 0.05), *** *p* < 0.001, ** *p* < 0.01, and * *p* < 0.05. Abbreviations: Nav1.1 (SCN1A)—sodium channel protein type 1 subunit alpha; Nav1.2 (*SCN2A)*—sodium channel protein type 2 subunit alpha; Cav1.2 (CACNA1C)—calcium voltage-gated channel subunit alpha1 C; Cav1.3 (CACNA1D)—calcium voltage-gated channel subunit alpha 1D subunit; Cav3.1 (CACNA1G)—calcium voltage-gated channel subunit alpha1 G; Cav3.2 (CACNA1H)—calcium voltage-gated channel subunit alpha1 H; Cav3.3 (CACNA1I)—calcium voltage-gated channel subunit alpha1 I; Kir4.1 (KCNJ10)—potassium inwardly rectifying channel subfamily J member 10; Ki67 (MKI67)—marker of proliferation Ki-67; BKCa A1 (KCNMA1)—potassium calcium-activated channel subfamily M alpha 1; BKCa B1 (KCNAB1)—potassium voltage-gated channel subfamily A regulatory beta subunit 1; BKCa B4 (Kcnma1)—potassium calcium-activated channel subfamily M alpha 1; TRPC3— transient receptor potential cation channel subfamily C member 3; TRPC7—transient receptor potential cation channel subfamily C member 7; Gabra (GABRA1)—gamma-aminobutyric acid type A receptor subunit alpha1; Gabbr (GABBR2)—gamma-aminobutyric acid type B receptor subunit 2; Nkcc1 (*SLC12A1)*—Na–K–Cl cotransporter isoform 1; Nkcc2 (*SLC12A2)*—Na–K–Cl cotransporter isoform 2; Gria1—glutamate ionotropic receptor AMPA type subunit 1; Gria2—glutamate ionotropic receptor AMPA type subunit 2; Grin2a—glutamate ionotropic receptor NMDA type subunit 2A; Grin2b—glutamate ionotropic receptor NMDA type subunit 2B; KA1 (GRIK4)- glutamate ionotropic receptor kainate type subunit 4; KA2 (GRIK5)—glutamate ionotropic receptor kainate type subunit 5; Pkca (PRKCA)—protein kinase C alpha; Pkce (PRKCE)—protein kinase C epsilon; Pik3ca (PIK3CA)—phosphatidylinositol-4,5-bisphosphate 3-kinase catalytic subunit alpha; Pik3cb (PIK3CB)—phosphatidylinositol-4,5-bisphosphate 3-kinase catalytic subunit beta; Pik3cg (PIK3CG)—phosphatidylinositol-4,5-bisphosphate 3-kinase catalytic subunit gamma.

At the age of 1 month, in the cortexes of the G9-1 mutant mice, an increase in the expression of the genes Cav 3.1, Cav 3.2, Cav 3.3, TRPC3, and TRPC7 was observed, and the expression of the genes Kir 4.1, Ki 67, BKCA1, and BKCB4 decreased (Figure 5A—black bars). In the cerebral cortexes of the A9-2 mutants at the age of 1 month, there was an increase in the expression of the BKCB4 and TRPC7 genes, and the expression of Cav 1.2, Cav 3.1, Kir 4.1, Ki 67, and BKCA1 decreased (Figure 6A—red bars). In the S5-1 mutants, only the Cav 3.3 expression increased. All the other tested genes encoding membrane channels either were decreased relative to the WT mice or did not change their expression (Figure 6A, blue bars). The expression levels of the Grin2a and Grin2b genes encoding the NMDAR subunits increased in all three strains (Figure 6B). As for the genes encoding other receptors, in the cerebral cortexes of the G9-1 mutants, there was an increase in Nkcc2, Gria1, and KA1, and the expression of Gabra and Nkcc1 decreased (Figure 6B, black bars). In the A9-2 mutants, the expression of Gabbr, Nkcc2, and Gria2 increased, and the level of Gria1 decreased (Figure 6B, red bars). In the cortexes of the S5-1 mutants, there was also an increase in the levels of Gabbr and Gria2, while the expression of the other genes tested did not change (Figure 6B, blue bars). On the other hand, for the genes encoding different isoforms of protein kinase A and PI3K, a decrease in all these genes was observed exclusively for the G9-1 mutants (Figure 6C, black bars), while for the other two mutant mice there was no change relative to the WT mice. In the cortexes of the A9-2 mutants, there was almost a 6-fold increase in the expression level of Pik3cg (Figure 6C, red bars).

**Figure 7:**
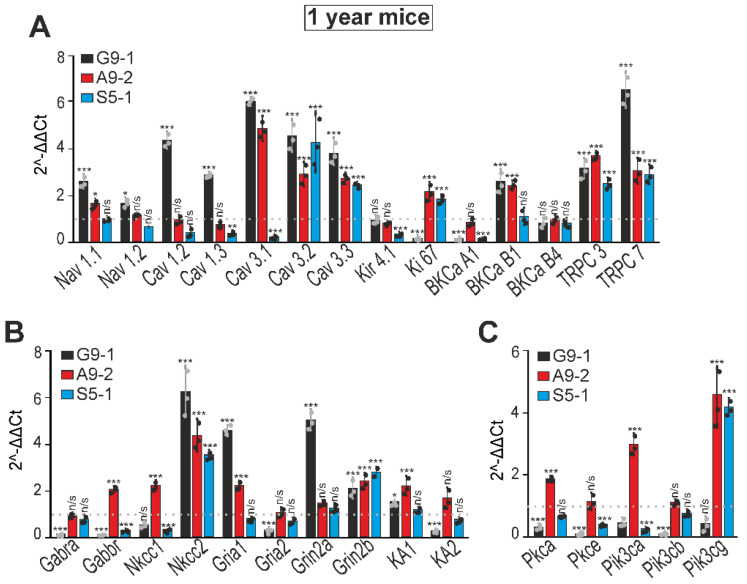
Expression patterns of genes encoding membrane ion channels (**A**), GABA and glutamate receptors (**B**), and protein kinases (**C**) in the cerebral cortexes of G9-1, A9-2, and S5-1 mice at the age of 1 year. 1 is the dotted line; the level of expression in the cerebral cortexes of WT mice is taken. Total RNA was obtained from cortex tissue of 1-year-old mice. Results are presented as mean ± SEM. Black asterisks indicate the differences between the experimental groups comparable to the WT mice group. n/s—data not significant (*p* > 0.05), *** *p* < 0.001, ** *p* < 0.01, and * *p* < 0.05. Abbreviations: Nav1.1 (SCN1A)—sodium channel protein type 1 subunit alpha; Nav1.2 (*SCN2A)*—sodium channel protein type 2 subunit alpha; Cav1.2 (CACNA1C)—calcium voltage-gated channel subunit alpha1 C; Cav1.3 (CACNA1D)—calcium voltage-gated channel subunit alpha 1D subunit; Cav3.1 (CACNA1G)—calcium voltage-gated channel subunit alpha1 G; Cav3.2 (CACNA1H)—calcium voltage-gated channel subunit alpha1 H; Cav3.3 (CACNA1I)—calcium voltage-gated channel subunit alpha1 I; Kir4.1 (KCNJ10)—potassium inwardly rectifying channel subfamily J member 10; Ki67 (MKI67)—marker of proliferation Ki-67; BKCa A1 (KCNMA1)—potassium calcium-activated channel subfamily M alpha 1; BKCa B1 (KCNAB1)—potassium voltage-gated channel subfamily A regulatory beta subunit 1; BKCa B4 (Kcnma1)—potassium calcium-activated channel subfamily M alpha 1; TRPC3— transient receptor potential cation channel subfamily C member 3; TRPC7—transient receptor potential cation channel subfamily C member 7; Gabra (GABRA1)—gamma-aminobutyric acid type A receptor subunit alpha1; Gabbr (GABBR2)—gamma-aminobutyric acid type B receptor subunit 2; Nkcc1 (SLC12A1)—Na–K–Cl cotransporter isoform 1; Nkcc2 (SLC12A2)—Na–K–Cl cotransporter isoform 2; Gria1—glutamate ionotropic receptor AMPA type subunit 1; Gria2—glutamate ionotropic receptor AMPA type subunit 2; Grin2a—glutamate ionotropic receptor NMDA type subunit 2A; Grin2b—glutamate ionotropic receptor NMDA type subunit 2B; KA1 (GRIK4)- glutamate ionotropic receptor kainate type subunit 4; KA2 (GRIK5)—glutamate ionotropic receptor kainate type subunit 5; Pkca (PRKCA)—protein kinase C alpha; Pkce (PRKCE)—protein kinase C epsilon; Pik3ca (PIK3CA)—phosphatidylinositol-4,5-bisphosphate 3-kinase catalytic subunit alpha; Pik3cb (PIK3CB)—phosphatidylinositol-4,5-bisphosphate 3-kinase catalytic subunit beta; Pik3cg (PIK3CG)—phosphatidylinositol-4,5-bisphosphate 3-kinase catalytic subunit gamma.

At the age of 1 year, in the cortexes of the G9-1 mutants, increased expression of the Nav 1.1, Cav 1.2, Cav 1.3, Cav 3.1, Cav 3.2, Cav 3.3, BKCB1, TRPC3, and TRPC7 genes was observed (Figure 7A, black bars), and the levels of Ki genes 67 and BKCA1 were downregulated. In the cortexes of the A9-2 mutants, the expression levels of Cav 3.1, Cav 3.2, Cav 3.3, Ki 67, BKCB1, TRPC3, and TRPC7 increased, and the levels of the other genes were at the level of the WT cells (Figure 7A, red columns). In the S5-1 mutants, increased levels of Cav 3.2, Cav 3.3, Ki 67, TRPC3, and TRPC7 genes were detected, and the expression of Cav 1.3, Cav 3.1, Kir 4.1, and BKCA1 was reduced (Figure 7A, blue bars). The expression of the Nkcc2, Gria1, Grin2a, and Grin2b genes was increased in the cortexes of the G9-1 mutants, and the levels of Gabra, Gabbr, Gria2, and KA2, on the contrary, decreased (Figure 7B, black bars). In the cortexes of the one-year-old A9-2 mice (Figure 7B, red bars), the expression of the Nkcc1, Nkcc2, Gria1, Grin2b, and KA1 genes was increased, and the expression of the other neurotransmission genes remained at the WT level. In the cortexes of the S5-1 mice, only the Nkcc2 and Grin2b expression increased, while the levels of Gabbr and Nkcc1 decreased (Figure 7B, blue bars). The level of expression of the genes encoding PKC and PI3K also decreased in the cortexes of the one-year-old G9-1 mice (Figure 7C, black bars), while, in the cortexes of the A9-2 mutants, on the contrary, there was an increase in the expression of these genes (Figure 7C, red bars). In the S5-1 mutants, there was a decrease in the Pkca and Pkce genes encoding PKC isoforms, and for PI3K there was a suppression of the Pik3ca gene, occurring with a four-fold increase regarding the Pik3cg gene (Figure 7C, blue bars).

Thus, in the cortexes of the G9-1 mutants, an increased level of expression of those genes encoding the protein channels of the neuronal cytoplasmic membrane, responsible for the Ca^2+^ ions’ transport into cells (L-type calcium channel, TRPC3- and TRPC7-channels, and calcium-activated BK channels) is observed throughout development (in 1 year of observation, the subunits of the NMDA receptors and the expression of the Gabra gene, encoding the inhibitory GABA (A) receptor, decreased sharply by 1 month of life and further in a year). Also, in the G9-1 mice, a significant and stable decrease in the expression of the genes encoding protein kinase C and phosphoinositol 3-kinase was observed. As for the A9-2 and S5-1 mutants, a low level of expression of most of the studied genes encoding the membrane protein transporters of the Ca^2+^ ions in the newborn state was established for them. With adulthood, the expression of these genes increased to the level of wild-type mice at 1 month, but, by one year, the expression of L-type calcium channels TRPC3 and TRPC7 became higher than in the WT mice. Also, for the newborn A9-2 and S5-1 mutants, a low level of expression of the genes encoding excitatory glutamate receptor channels and inhibitory GABA receptors was established, but by 1 month the expression of especially the NMDA receptors reached elevated levels relative to the cortexes of the wild-type mice. As for the genes encoding PKC and PI3K, it was reduced in the newborn cortexes of the A9-2 and S5-1 mutants but reached the expression level of the wild-type mice already by 1 month of mouse development. Thus, the resulting mutant mouse lines exhibit different patterns of expression of the genes encoding the proteins of neurotransmission and signaling, where the G9-1 mutants are characterized by persistently increased expression of a number of genes and the A9-2 and S5-1 mutants have a predominantly reduced level of gene expression relative to the wild type in the newborn cortex, but, by 1 month or 1 year, the expression of most genes reaches elevated levels.

### 3.4. Ca^2+^ Activity of Excitatory Ionotropic Glutamate Receptors Is Changed in the Cortical Neurons of Mutant Strains

**Figure 8:**
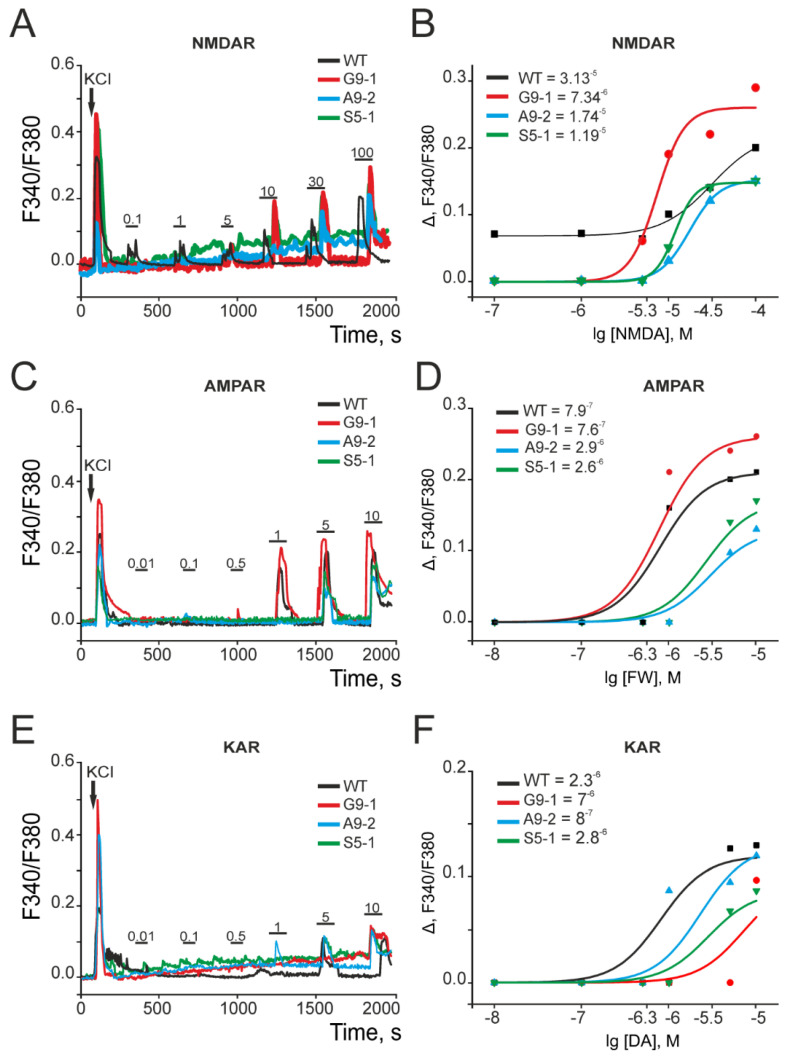
Effect of ENU-directed mutagenesis on Ca^2+^ signals of cortical neurons upon activation of excitatory ionotropic glutamate receptors. (**A**)—Application of different concentrations of NMDA in Mg^2+^-free medium to the cortical neurons isolated from the WT, G9-1, A9-2, and S5-1 mouse lines. (**C**)—Application of different concentrations of 5-Fluorowillardiine (FW) in the presence of an inhibitor for desensitization of AMPA receptor cyclothiazide (CTZ, 5 µM) to the cortical neurons isolated from the WT, G9-1, A9-2, and S5-1 mouse strains. (**E**)—Application of different concentrations of KA receptor activator domoic acid (DA) in the presence of a selective antagonist of AMPA receptor GYKI-52466 (30 µM) and an inhibitor for desensitization of KA receptor concanavalin A (ConA, 200 µg/mL) to the cortical neurons isolated from the WT-, G9-1-, A9-2, and S5-1 mouse strains. NMDA, FW, and DA concentrations (μM) are specified with horizontal lines. The average Ca^2+^ signals of neurons are presented. (**B**,**D**,**F**)—Dependence of the Ca^2+^ responses’ amplitudes on NMDA (**B**), FW (**D**), and DA (**F**) concentrations in the cortical neurons from WT, G9-1, A9-2, and S5-1 mutants. F340/F380—Fura-2 fluorescence in arbitrary units as a result of dividing the 340 nm recording channel by the 380 nm probe recording channel. Δ, F340/F380—average amplitudes of neuronal Ca^2+^ signals, expressed in arbitrary units of Fura-2 fluorescence, as a result of subtracting the base fluorescence of the probe from the maximum Ca^2+^ signal of cells during applications of receptor activators (Δ = Fmax–Fmin).

We hypothesized that such multidirectional and pronounced changes in the expression patterns of those genes encoding the proteins involved in neurotransmission should be manifested in differences in neuronal intracellular signaling. One such sign is the [Ca^2+^] concentration. In order to test this, we measured the [Ca^2+^] concentrations in the primary cell cultures isolated from the mutant and wild type brains. The primary cortical neuroglial cultures were isolated from newborn mice. The cells were loaded with the Ca^2+^-sensitive probe Fura-2, and increases in the [Ca^2+^]_i_ were recorded in response to the selective activators of the key excitatory ionotropic glutamate receptors—NMDAR, AMPAR, and KAR. The Fura-2 probe is ratiometric, and the fluorescence was recorded in two wavelengths: 340 nm (Ca^2+^-bound form of Fura-2) and 380 nm (Ca^2+^-free form), and the result of dividing the 340 nm channel by 380 nm (F340/F380) reflects the changes in the intracellular concentration of Ca^2+^. The application of increasing concentrations of NMDA was carried out for 30 s in a magnesium-free (Mg^2+^-free) medium, after which the activator was washed away with a complete medium substitution. The neurons isolated from the WT mice cortexes began to respond with an [Ca^2+^]_i_ increase to the application of 0.1 μM NMDA (Figure 8A, black curve), and an increase in the concentration of the agonist led to an increase in the amplitude of the Ca^2+^ signal (Figure 8B). The cortical neurons of the G9-1 mutants began to respond with an [Ca^2+^]_i_ increase at an NMDA concentration of 5 μM and higher (Figure 8A, red curve). At the same time, the cortical neurons from the A9-2 and S5-1 mutants responded with small [Ca^2+^]_i_ increases upon the application of 10 μM NMDA (Figure 8A). The analysis of the Ca^2+^ signals’ amplitude dependences on the concentration of NMDA (reflects the EC50 value) showed that the EC_50_ for the WT neurons was 31.3 µM, for the G9-1 neurons 7.3 µM, and for the A9-2 and S5-1 neurons 17.4 and 11.9 µM, respectively (Figure 8B).

To activate the AMPARs, we used the selective agonist 5-Fluorowillardiine (FW), which was applied to neurons in the presence of an AMPAR desensitization inhibitor, cyclothiazide (CTZ, 5 µM). The application of FW at a concentration of 1 µM caused an [Ca^2+^]_i_ increase in the WT and G9-1 neurons (Figure 8C, black and red curves), while, in the A9-2 and S5-1 neurons, the Ca^2+^ signals were recorded with a 5 µM agonist (Figure 8C, green and blue curves). The resulting EC_50_ values upon the activation of the AMPARs were 790 nM, 760 nM, 2.9 μM, and 2.6 μM for the WT, G9-1, A9-2, and S5-1 neurons, respectively (Figure 8D).

To activate the KARs, we used the agonist domoic acid (DA) in the presence of a selective antagonist of the AMPA receptors, GYKI-52466 (30 µM), and an inhibitor for the desensitization of the KA receptors, concanavalin A (ConA, 200 µg/mL). The Ca^2+^ signals of the WT and S5-1 neurons to the KAR activator were recorded with a 5 µM agonist (Figure 8E, black and green curves), the cortical neurons from the G9-1 mutants responded at 10 µM DA (Figure 8E, red curve), and the A9-2 neurons responded with Ca^2+^ signals upon the application of 1 μM DA (Figure 8E, blue curve). At the same time, for the KAR agonist, the EC_50_ values were 2.3 μM, 7 μM, 8 μM, and 2.8 μM for the WT, G9-1, A9-2, and S5-1 neurons, respectively (Figure 8F).

Thus, differences in the activity of excitatory glutamate receptors have been established for the neurons isolated from all three mutant strains. It turned out that the cortical neurons of the mutant mice are characterized by reduced sensitivity to NMDAR activation relative to the WT neurons. However, at high concentrations of the agonist, the highest amplitudes of Ca^2+^ signals were recorded in the G9-1 neurons. As for the AMPARs, the G9-1 mutant neurons showed greater sensitivity to the activator, but the EC_50_ value was close to the WT neurons. The least-sensitive to NMDAR and AMPAR activation were the S5-1 and A9-2 neurons, respectively. In the case of KARs, the A9-2 neurons turned out to be more sensitive to domoic acid, requiring almost two times less agonist concentration for activation compared to the WT neurons. And, the G9-1 mice turned out to be the least sensitive to KAR activation.

### 3.5. Characteristics of Spontaneous and Epileptiform Ca^2+^ Activity of Cortical Neurons Obtained from G9-1, A9-2, and S5-1 Mice

**Figure 9:**
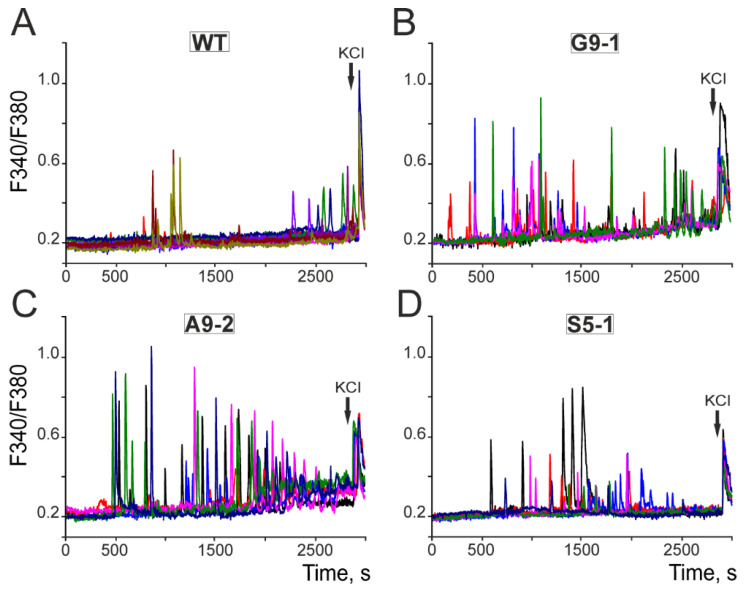
Characteristics of spontaneous Ca^2+^ activity of neurons obtained from WT mice (**A**), G9-1 (**B**), A9-2 (**C**), and S5-1 (**D**) mutants. To identify neurons, 35 mM potassium chloride (KCl) was applied at the end of the experiment. Typical Ca^2+^ signals of neurons are presented. Cell culture age 14 DIV. F340/F380—Fura-2 fluorescence in arbitrary units as a result of dividing the 340 nm recording channel by the 380 nm probe recording channel.

**Figure 11:**
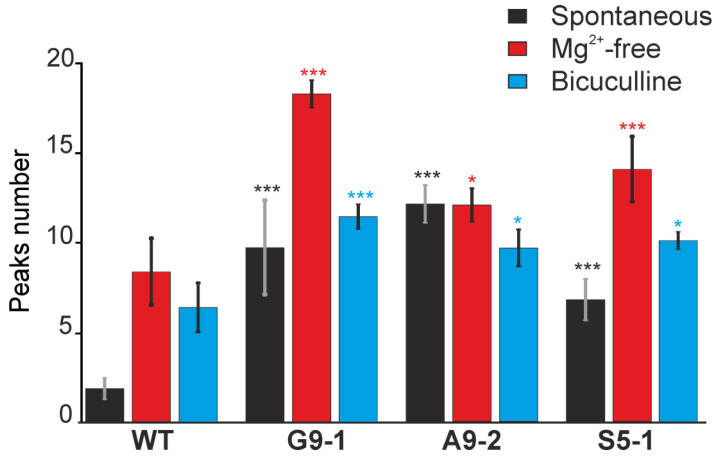
Analysis of the frequency of spontaneous, induced by magnesium-free (Mg^2+^-free) medium or inhibition of GABA-A receptors (bicuculline 10 μM), Ca^2+^ impulses in neurons isolated from WT mice, G9-1, A9-2, and S5-1 mutants. Averaged results obtained on 6 cell cultures are presented. The presented data correspond with the results presented in Figure 9 and Figure 10. Results are presented as mean ± SEM. Black asterisks indicate the differences between the experimental groups comparable to the WT group. *** *p* < 0.001 and * *p* < 0.05.

Differences in the sensitivity of excitatory glutamate receptors to agonists may contribute to the formation of epileptiform neuronal activity. To study the spontaneous neuronal Ca^2+^ activity, the cell cultures were loaded with the Ca^2+^-sensitive probe Fura-2, mounted on a fluorescence microscope, and the generation of spontaneous Ca^2+^ signals in the neurons was recorded for 45 min. To identify the neurons, 35 mM KCl was added at the end of the experiments (Figure 9). In the culture of the WT neurons, single Ca^2+^ pulses were recorded in the individual cells and, as a rule, closer to the end of the recording time (Figure 9A), with an average frequency of 1.9 ± 0.5 pulses during the recording time (Figure 11). In the G9-1 (Figure 9B), A9-2 (Figure 9C), and S5-1 neurons (Figure 9D), the generation of asynchronous Ca^2+^ impulses and Ca^2+^ oscillations of various amplitudes was recorded. The frequency of the Ca^2+^ impulses for the G9-1 neurons was 9.75 ± 2.9 impulses and for the A9-2 and S5-1 neurons 12.2 ± 1.1 and 6.8 ± 1.1 impulses (Figure 11), respectively.

**Figure 10:**
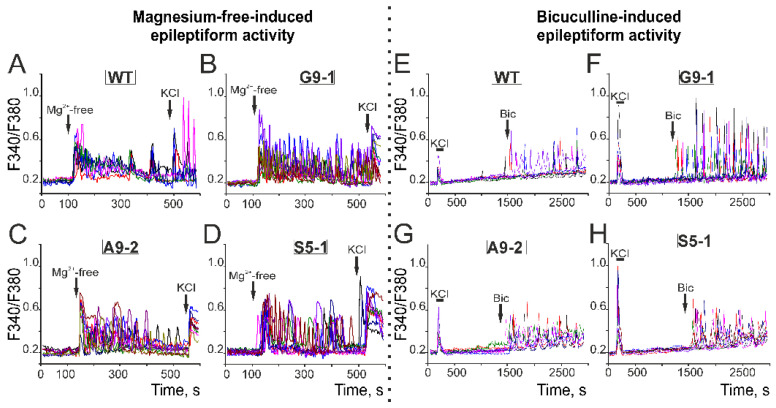
Characteristics of Ca^2+^ activity of neurons obtained from WT mice (**A**,**E**), G9-1 (**B**,**F**), A9-2 (**C**,**G**), and S5-1 (**D**,**H**) mutants when modeling epileptiform activity by excluding Mg^2+^ ions from the medium (Mg^2+^-free, magnesium-free-induced epileptiform activity) or using GABA(A) receptor inhibition upon application of 10 µM bicuculline (Bic, bicuculline-induced epileptiform activity). To identify neurons, 35 mM potassium chloride (KCl) was applied at the end or beginning of the experiment. Typical Ca^2+^ signals of neurons are presented. Cell culture age 10 DIV. F340/F380—Fura-2 fluorescence in arbitrary units as a result of dividing the 340 nm recording channel by the 380 nm probe recording channel.

Modeling the neuronal epileptiform activity by excluding Mg^2+^ ions from the extracellular environment led to the generation of Ca^2+^ signals in both the WT neurons and neurons isolated from the cerebral cortexes of the mutant mice (Figure 10). In general, all the neurons are characterized by a rapid synchronous [Ca^2+^]_i_ increase upon the application of a Mg^2+^-free medium, followed by asynchronous Ca^2+^ pulses of various frequencies and amplitudes. The WT neurons are characterized by low-amplitude Mg^2+^-free-induced Ca^2+^ impulses (Figure 10A) with an average frequency of 8.3 ± 2.6 impulses during recording (Figure 11). In the G9-1 neurons, a higher amplitude of Ca^2+^ oscillations occurred in response to Mg^2+^-free (Figure 10B), with an average frequency of 19.2 ± 1.2 impulses (Figure 11). In the A9-2 neurons (Figure 10C) and S5-1 neurons (Figure 10D), the Mg^2+^-free-induced Ca^2+^ signals also represent predominantly high-amplitude Ca^2+^ oscillations, occurring with frequencies of 12.9 ± 0.9 and 14.7 ± 1.8 impulses (Figure 11), respectively.

The second in vitro model of epileptiform activity that we used to characterize the neurons isolated from the cortexes of the mutant mice is based on blocking inhibitory GABA(A) receptors using the application of 10 μM bicuculline (Figure 10, Bicuculline-induced epileptiform activity). In the WT neurons, bicuculline most often led to the appearance of single Ca^2+^ pulses, but, in some neurons, it led to asynchronous Ca^2+^ oscillations (Figure 10E) with an average pulsation frequency of 6.4 ± 1.4 pulses (Figure 11). In the cortical neurons of the G9-1 mutants, the application of bicuculline led to the induction of high-amplitude Ca^2+^ oscillations with a high degree of synchronization (Figure 10F) and an average frequency of 10 ± 1.5 (Figure 11). In the A9-2 (Figure 10G) and S5-1 (Figure 10H) neurons, Ca^2+^ oscillations also occurred in response to bicuculline but with lower amplitudes compared to the G9-1 neurons and frequency values of 9.7 ± 1.0 and 10.5 ± 0.5 pulses during registration (Figure 11), respectively.

Thus, the neurons isolated from the cerebral cortexes of all three ENU mutant lines showed increased spontaneous Ca^2+^ activity compared to the WT neurons. The G9-1 neurons showed the most pronounced frequency and amplitude of Ca^2+^ signals. In the models of epileptogenesis, the neurons obtained from the mutant mice were also more excitable when a magnesium-free medium or blocking of GABA(A) receptors caused high-amplitude, high-frequency Ca^2+^ oscillations.

## 4. Discussion

Animal models proved to be very useful to investigate the molecular and cellular mechanisms of neurological diseases, including epilepsy. Pharmacological models like injections of pilocarpine or kainate in a mouse with further modeling of audiogenic epilepsy or electrical stimulation data regarding acquired epilepsy demonstrated high potential in antiseizure drug screening [25]. However, a significant number of epilepsies are genetically determined and strongly resistant to therapy. Using genetically modified mice for “knockout” targeting of specific genes, which is very useful, at the same time poses some limitations for studying the mechanisms of epileptogenesis and developing effective antiseizure drugs for genetically determined seizures. Indeed, most of the classical knockout models represent gene “loss-of-function” models. In the case of the complete deletion of the targeted gene, these animals often have a lethal phenotype or severe phenotypes due to disturbances in other molecular pathways associated with the targeted gene, which is not related to epileptogenesis [26]. It has been shown that a significant number of neurological diseases, including epilepsy, are caused by single-nucleotide gene mutations that disrupt protein function in a subtler way than direct inactivation [27]. ENU-directed mutagenesis is an effective and appropriate tool for modeling such cases of epileptogenesis since it enables generating point mutations of genes [16]. The generation of mouse mutant strains by ENU mutagenesis enables the generation of mutants that can model such forms of epilepsy in order to study the mechanisms of genetically determined epileptogenesis and use them for drug screening. ENU-directed mutagenesis allowed us to generate three novel mouse strains that are susceptible to seizures and characterized by behavioral and cognitive dysfunctions. Interestingly, the G9-1 and S5-1 mice showed the most pronounced cognitive and behavioral disorders, manifested in increased anxiety, poor learning ability, and reduced motor activity, together with epileptic phenotypes such as «wild run» and seizures after audiogenic stimulation. In the A9-2 mice, increased anxiety and reduced motor activity were also detected. However, the learning ability was preserved and there were no signs of epilepsy after audiogenic stimulation in the late backcrossed generations. 

The use of electroencephalography for recording epileptic states is generally accepted and indicative [28]. An encephalogram is capable of revealing several patterns of epileptic activity of the brain network, including spikes, polyspikes, waves, hypsarrhythmia, paroxysmal bursts, etc. [29]. It has been shown that the amplitude and frequency of electrical oscillations result from a sequence of processes of inhibition and excitation of the postsynaptic potentials of pyramidal neurons [30,31,32]. In turn, the dynamics of the Ca^2+^ ions in the cytosol of neurons drive the synaptic activity [33,34]. It is known that an [Ca^2+^]_i_ increase in neurons induces the release of excitatory neurotransmitters, just as in brain injuries an increase in glutamate promotes the activation of excitatory glutamate receptors. An [Ca^2+^]_i_ increase may be associated, under certain conditions, but not always, with epileptic seizures [35,36]. In the present study, we did not record the eclectic activity of the cerebral cortex using electroencephalography, but the use of calcium neuroimaging is one of the widely used approaches for detecting epileptiform activity [37,38]. We managed to establish several patterns of “behavior” of the Ca^2+^-signaling system of neurons obtained from the cerebral cortexes of mutant mice. In particular, the measurements of the Ca^2+^ dynamics showed that the cortical neurons obtained from different mutant strains during the modeling of epileptiform activity generate both single Ca^2+^ spikes of different amplitudes and Ca^2+^ oscillations of different frequencies, which are at least good characteristics of the obtained mutants at this stage of the research.

**Figure 12:**
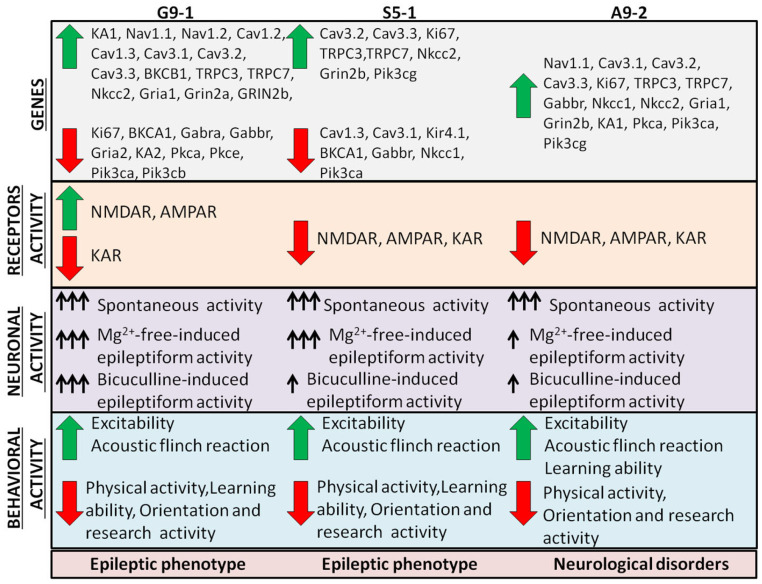
Summary of the ENU-directed mutagenesis effects on the expression of genes encoding neurotransmission proteins, the activity of excitatory ionotropic glutamate receptors, and neuronal activity in cortical neurons, as well as the behavioral characteristics of ENU mutant strains. Green arrows indicate increasing effects, and red arrows indicate decreasing effects. Black arrows indicate rate of neuronal activity increase. Abbreviations: KA1 (GRIK4)—glutamate ionotropic receptor kainate type subunit 4; KA2 (GRIK5)—glutamate ionotropic receptor kainate type subunit 5; Nav1.1 (SCN1A)—Sodium channel protein type 1 subunit alpha; Nav1.2 (*SCN2A)*—Sodium channel protein type 2 subunit alpha; Cav1.2 (CACNA1C)—calcium voltage-gated channel subunit alpha1 C; Cav1.3 (CACNA1D)—calcium voltage-gated channel subunit alpha 1D subunit; Cav3.1 (CACNA1G)—calcium voltage-gated channel subunit alpha1 G; Cav3.2 (CACNA1H)—calcium voltage-gated channel subunit alpha1 H; Cav3.3 (CACNA1I)—calcium voltage-gated channel subunit alpha1 I; BKCa B1 (KCNAB1)—potassium voltage-gated channel subfamily A regulatory beta subunit 1; BKCa A1 (KCNMA1)—potassium calcium-activated channel subfamily M alpha 1; TRPC3— transient receptor potential cation channel subfamily C member 3; TRPC7—transient receptor potential cation channel subfamily C member 7; Nkcc1 (*SLC12A1)*—Na–K–Cl cotransporter isoform 1; Nkcc2 (*SLC12A2)*—Na–K–Cl cotransporter isoform 2; Gria1—glutamate ionotropic receptor AMPA type subunit 1; Gria2—glutamate ionotropic receptor AMPA type subunit 2; Grin2a—glutamate ionotropic receptor NMDA type subunit 2A; Grin2b—glutamate ionotropic receptor NMDA type subunit 2B; Ki67 (MKI67)—marker of proliferation Ki-67; Gabra (GABRA1)—gamma-aminobutyric acid type A receptor subunit alpha1; Gabbr (GABBR2)—gamma-aminobutyric acid type B receptor subunit 2; Pkca (PRKCA)—protein kinase C alpha; Pkce (PRKCE)—protein kinase C epsilon; Pik3ca (PIK3CA)—phosphatidylinositol-4,5-bisphosphate 3-kinase catalytic subunit alpha; Pik3cb (PIK3CB)—phosphatidylinositol-4,5-bisphosphate 3-kinase catalytic subunit beta; Pik3cg (PIK3CG)—phosphatidylinositol-4,5-bisphosphate 3-kinase catalytic subunit gamma; Kir4.1 (KCNJ10)—potassium inwardly rectifying channel subfamily J member 10; NMDAR—N-methyl-D-aspartate receptor; AMPAR—α-amino-3-hydroxy-5-methyl-4-isoxazolepropionic acid receptor; KAR—kainic acid receptor; ↑, ↑↑↑—demarcate Ca^2+^-concentration of neurons modulating epileptiform activity in vitro, estimated by the frequency and amplitude of Ca^2+^-oscillations.

An important component of cognitive dysfunction in epilepsy is altered neural networks [26]. At the organism level, it was found that, in children with epilepsy, the neural networks were characterized by delays in neuronal differentiation and disruption of the connections between certain areas of the brain [27]. Seizures often develop at the level of the neuroglial complex. The “epileptic” neurons are often characterized by the instability of the membrane, a tendency towards depolarization, and the lability of the membrane potential. An epileptic focus is a group of neurons with pathological electrogenesis that generate excessive neuronal discharges, sometimes leading to the hypersynchronization of the surrounding neurons. It is known that one of the key mechanisms in the development of epilepsy is an imbalance between the excitation and inhibition of the neuroglial networks [39]. The excitation of the networks occurs primarily due to glutamate, which acts through NMDARs, AMPARs, and KARs, causing rapid depolarization of the postsynaptic cell membranes of neurons. This process of depolarization and propagation of excitation lasts until astrocytes capture glutamate from the synaptic cleft and process it into glutamine [35]. Using microdialysis, it was discovered that increased concentrations of glutamate were found in patients with epilepsy not only during seizures but also preceding their generation, which confirms the role of the hyperactivation of the glutamatergic neuronal system in the occurrence and propagation of epileptic seizures [39,40,41]. At the same time, the inhibitory neurotransmitter GABA is released. Before the seizure, the GABA release transients were weakening, while the glutamate transients were increasing, indicating a failure of the network inhibition [42]. The neurophysiological mechanisms associated with epileptogenesis are influenced by genes that control various biochemical processes, such as receptor activity and neurotransmitter interactions [43]. We were able to demonstrate that the G9-1 mutants caused dramatic changes in the expression of the genes encoding excitatory glutamate receptors. These mutants showed increases in the expression of the genes encoding excitatory NMDARs, AMPARs, and KARs (Figure 12). At the same time, in the G9-1 mutant neurons, there were decreases in the expression of the inhibitory GABA receptors and protein kinases (PI3K and PKC), which are considered to be neuroprotective. In the S5-1 and A9-2 mutants, the changes in the expression of the genes encoding the receptors of the excitatory component of neurotransmission were less pronounced; in the cortexes of both mice lines, there were increases in the expression of the Grin2b gene encoding the NMDAR subunit.

Genome-wide studies demonstrate a polygenic pattern of inheritance and the presence of multiple single-nucleotide polymorphisms, accounting for about 58% of the genetic changes in idiopathic generalized epilepsies [44]. The changes in the gamma-aminobutyric acid receptor genes GABRA1 (rs2279020), GABRG2 (rs121909673), and GABRA6 (rs3219151) are widely known [7,45]. In patients with juvenile myoclonic epilepsy, several individual loci are involved, of which GABRA1, GABRD, EFHC1, BRD2, CASR, and ICK with complex inheritance are considered to be the most pathogenic [46]. In the S5-1 mutants, the expression of the gene encoding the GABA-B receptor is decreased, while, in the A9-2 mice, on the contrary, this receptor expression is increased. In all three lines of mutant mice, an imbalance in the expression of the excitatory and inhibitory receptors responsible for neurotransmission and epileptogenesis was detected, but the imbalance was of a differential nature. It is important to distinguish between the changes in the GABAergic neurotransmission in the cases of genetically determined epilepsy and acquired epilepsy. It was shown that there was a decrease in the number of GABA-positive neurons in the cortex when epilepsy was induced by cobalt administration [47]. Also, in a monkey model of epilepsy injected with alumina gel, a decrease in the number of GAD-positive axon terminals was observed [48]. Rats treated with alumina also experienced a progressive loss of GAD-positive neurons, which correlated with the development of abnormal cortical activity and behavioral seizures [49]. However, as for some genetically determined epilepsies, on the contrary, increases in the number of GABA neurons have been established. In El (epileptic) mice, an increase in the number of GABA-immunoreactive neurons was detected [50]. Similar trends were found in genetically epilepsy-prone (GEPR) rats [51] and in seizure-susceptible gerbils [52]. In such genetic models of epilepsy, increased numbers of GABAergic neurons and inhibitory neurotransmission are hypothesized to have a synchronizing effect that results in a paradoxical improvement in some types of epileptic seizures. Thus, in the G9-1 mice, there was a trend toward an increase in the number of GABAergic neurons in the cerebral cortex, even without division into groups with epileptiform activity or wild run, while in the A9-2 line (without an epileptic phenotype) or S5-1, no increase was detected in the number of GABA-immunoreactive neurons compared to the control group (Balb/c). At the same time, in the cortical slices of the G9-1 mice in the group with an epileptic phenotype, a significant decrease in GABA-positive neurons was revealed, and, in the S5-1 line, on the contrary, an increase in the number of GABA-positive neurons was detected both in the “wild run” group and in the “epilepsy” group. Such discrepancies in the experimental data are descriptive of the characteristics of the generated mutants, and, in this study, we do not aim to describe or compare the inhibitory component of neurotransmission. However, there is compelling evidence in the literature regarding attempts to describe epilepsy simply as a failure of GABAergic inhibition, while other authors suggest that hyperinhibition masks hyperexcitation. We are inclined to believe that the “too much or too little GABA” paradigm does not work due to the complexity of the changes in the cortex after status epilepticus and the problems with the mechanistic understanding of epileptogenesis at this stage of the research, as shown in the work of Scharfman and Brooks-Kayal on the dentate fascia [53]. In addition, the GABAergic component of neurotransmission is characterized by extreme plasticity under noxious stimuli. GABA neurons are capable of exerting both suppressive and attenuating effects on seizure activity, exhibiting short-term or long-term effects. Those GABAergic neurons perform pleiotropic functions in epilepsy [54].

In addition, the accumulation of glutamate in the synaptic cleft in epilepsy leads to the depletion of glutamate receptors, activation of Na^+^ and Ca^2+^ channels, accumulation of Na^+^ and Ca^2+^ ions inside the cell, and K^+^ ions in the extracellular fluid. This in turn promotes the release of Ca^2+^ from the intracellular store and activation of enzymes (phospholipases, proteases, etc.), accumulation of arachidonic acid, increased lipid peroxidation, and destruction of cell membranes [35]. There is evidence that more than 950 genes are associated with the development of epilepsy. Most of these genes encode ion channels permeable to Na^+^, K^+^, Ca^2+^, Cl^−^, and other ions located in the neurolemma [55,56]. Using PCR analysis, we were able to establish that, in the G9-1 mutants, the expression of ten of the fourteen studied genes encoding Na^+^-, K^+^-, and Ca^2+^-conducting channels in the plasma membrane of neurons increased, whereas, in the S5-1 and A9-1 mutant mice, increased expression was recorded for four and seven genes, respectively (Figure 12). The general trend was an increase in the expression of those genes encoding the TRPC channel. Studies of the TRPC channel’s role in epileptiform activity have been conducted mainly regarding the hippocampus. However, the hippocampus is a part of the cerebral cortex, and these results can be extrapolated to our data. TRPC3 expression is believed to be increased in the hippocampi of rats with pilocarpine-induced epilepsy [57]. The intracerebroventricular infusion of the selective TRPC3 inhibitor compound Pyr3 protects the hippocampal pyramidal neurons after an epileptic seizure [57,58]. It is also known that global ablation of the TRPC3 channels markedly reduces the duration and severity of behavioral seizures in mice treated with pilocarpine, and also reduces the overall root mean square (RMS) power and theta activity of epileptic seizures [59]. It has been shown that the genetic deficiency of TRPC7 in mice significantly prevents the induction of epileptic seizures and reduces the mortality of animals after treatment with pilocarpine [60]. The role of Na^+^ channels in epileptogenesis has been well studied. There is a concept called sodium channelopathies, when impaired expression or mutation of the Na^+^ channel protein leads to disruption of the transport of sodium ions and changes the ability of neurons to generate/conduct a signal, which leads to epilepsy. Nav1.1 channels play a special role here [61], the expression of which increased in the cortexes of the G9-1 and A9-2 mutants. An imbalance in K^+^ ions is involved in epileptogenesis, when an excess of these ions in the extracellular space contributes to the generation of seizures. During intense electrical activity, the high extracellular concentration of Na^+^ does not change noticeably, but the concentration of K^+^ can increase significantly. Several types of K^+^ channels have been found in various cells, which differ in the parameters of the channel state depending on the membrane potential, intracellular Ca^2+^ content, and other factors [62]. In the cortexes of the G9-1 and S5-1 mice, which have an “epilepsy” phenotype, increased expression of the Ca^2+^-activated potassium channels (BK channels) was detected, while in the A9-2 mice these genes did not change, which may contribute to the development of the epileptic phenotype. According to genetic studies, the role of the genes encoding the calcium channels, CACNA1H, CACNA1G, and CACNG3, is shown in the etiopathogenesis of childhood absence epilepsy [63]. In the mutant mice of all three lines, there were increases in the expression of those genes encoding voltage-dependent Ca^2+^ channels, but the most pronounced effect of the mutation was detected in the G9-1 line.

All the above-described complex changes in the expression of the genes and proteins involved in the hyperexcitation of neuronal networks led to an increased propensity of neurons obtained from mutant mice to induce epileptiform activity in culture. It turned out that the cortical neurons isolated from all three mutant strains are characterized by increased spontaneous Ca^2+^ activity, but the highest frequency of Ca^2+^ oscillations was recorded in the G9-1 and S5-1 neurons. Similarly, in the magnesium-free and bicuculline in vitro models of epileptiform neuronal activity, the highest frequencies of Ca^2+^ oscillations were observed in the G9-1 neurons. For a number of models of genetically inherited neurological diseases, signs of spontaneous epilepsy in the absence of audiogenic epileptiform activity phenotypes have been shown. In the mouse model of Mowat–Wilson syndrome, which is caused by a Sip1 gene mutation, Rett syndrome, caused by an inactivation of MeCP2, and Satb2-associated syndrome, which occurs when the expression of transcription factor Satb2 is disrupted, audiogenic stimulation did not cause epileptic seizures. However, the neurons and astrocytes isolated from these mice in culture were highly prone to inducing hyperexcitation in in vitro models of epileptiform activity, and adult mice were characterized by numerous cognitive and behavioral deficits [64,65,66,67,68].

## 5. Conclusions

We utilized ENU-induced mutagenesis in order to generate novel mouse strains that are susceptible to seizures. We also characterized various aspects of their mutant phenotypes, such as behavior, cytoarchitecture, and the expression of the genes encoding the proteins involved in neurotransmission. In all three mouse strains, the severity of the behavioral disorders, changes in the expression of those genes encoding neurotransmission proteins during mouse cortex development, and the Ca^2+^-signaling system of the neurons correlate with the seizure severity. Although the genetic loci regarding these mutations have not been mapped, these strains will be useful tools for antiseizure drug testing in vivo.

## Figures and Tables

**Table 1:** Sequences of oligonucleotides used in the real-time PCR reaction.

Primers for the Synthesis of Mice mRNA Fragments		
Gene Name	Forward Primer 5′→3′	Reverse Primer 5′→3′
*GAPDH*	AAGGTGGTGAAGCAGGCATC	CTCTTGCTCAGTGTCCTTGC
*SELENOT*	TGATTGAGAACCAGTGTATGTC	GGTACAACGAGCCTGCCAAG
*SELENOM*	CGCCTAAAGGAGGTGAAGGC	CTTGCGGTAGAAGCCGAGCTC
*SELENOF*	AGGGTGCTGTCAGGAAGAAG	CGTTCCAACTTCTCGCTCAG
*SELENOK*	GAAGAGGGCCACCAGGAAAC	GGAATTCCCAGCATGACCTC
*SELENOS*	GGACCAAGCCGAGACTGTTC	CTTCTTGCATGCTGTCCCAC
*SELENON*	AAGATGGCTTCCTAGGGGTC	CTGAGGGGCAAAGCGGGTC
*SELENOH*	AAGGGCCCTCCACGAAAGC	TGGTGAGGAAGAAACTATGGC
*SELENOI*	CAGTGTTTTTGTTCTTTATCTCC	GGAAGAGTCACACATAATGCAC
*SELENOO*	CGTGCAGGCCCCAGCGTG	TTGGAAGAGTCACACATAATGC
*SELENOP*	CTGGACTCTCTTAAATGGAAAC	CCGCAGTTTTATTGGCAGTAG
*SELENOW*	GACAGTAGCCGGGAAGTTGG	GGAACATCGAGGAAAGACCAC
*SELENOV*	GGTAAGCTCTCCACGATTTCC	TTCAAACTCCCCTGTAACCTG
*TXNRD1*	CAACAAATGTTATGCAAAAATAATC	ACACTGGGGCTTAACCTCAG
*TXNRD2*	ACTATGGCTGGGAGGTGGC	CTCCAGTAGCAATGATGATGTG
*TXNRD3*	CCTTTGACTTGTGTGGGGTAC	CTCTTTAGAAAAGTGTGATTATATT
*DIO1*	ACAACGTGGACATCCGGCAG	GTGTCTAGGTGGAGTGCAAAG
*DIO2*	GCTTATCTCTGCCCCCATTG	CACACATAAACGACCTCCTTC
*DIO3*	CCCAGACCGCCTCGTGCC	CTCCTCGCCTTCACTGTTGA
*GPX1*	GGGGAGCCTGTGAGCCTGG	GGACGTACTTGAGGGAATTC
*GPX2*	CGCTTTCCCAGGCGCCTGG	GCTTGGGATCGGTCATGAGG
*GPX3*	GAAAGGAGATGTGAACGGGG	GTGGGGGCATCAGTTACTTC
*GPX4*	GATGAAAGTCCAGCCCAAGG	GAAGGCTCCAGGGGTCACAG
*Nav1.1*	TGGTGTTGGCCATCATTGTCTTTA	CAACGTGGGAGTTTGCAGTCAGT
*Nav1.2*	GCGTGCTGCTGGGAAAACATATA	GCCACCACAGCCAGGATCAA
*Cav 1.2*	CAAAGGCTACCTGGACTGGATCAC	AGCCACGTTTTCAGTGTTGACAGA
*Cav 1.3*	AAGACATCGATCCCGAGAACGAG	CACCAGCACCAGAGAGTTCCACA
*Cav 3.1*	CTACTTCGAAACCGATGCTTCCTC	CTCTCGTCCTCATTCTCTGTCTGGTA
*Cav 3.2*	AGGAACAACAACCTGACCTTCTTGC	AGCATTTCTGCATGCCGTTGTC
*Cav 3.3*	GACACATGGAACCGCTTGGACT	AGGCCTCAGGACACGCACAGT
*Kir4.1*	CACTGGCCATCGTGCTTCTTAT	GGCAATCTTTGCAAGGAAGGTAC
*Ki67*	GATGAGAAGCCTGTACCTGAG	GTTTTACATTGGTTTTCTTTCGG
*BKCa_A1*	GCCTCTTCATCATCTTGCTCTGG	TGGCTGGAGCCATTGTTTATCTT
*BKCa_B1*	CCCTCTACCAGAAAAGTGTGTGGAC	CTCCAGCTCTTCCTGGTCCTTGA
*BKCa_B4*	GAACTGGCAGCAGTATTGGAAAGAT	AGGAGAACGTCCTCTGGTCTCTGA
*TRPC3*	AGTCTCGCACGCTCAATGTCAAC	AGCTCGGTCACCTCCAGATGCT
*TRPC7*	CCGTGCAGCAAGCTAGGACAAAC	CGTTCACAACTAGCAGTCCCAAGAA
*Gabra*	TATCTTTGGGCCTGGACCCTCATTCTG	CCATAAGGTTGTTTAGCCGGAGCACTG
*Gabbr*	TCCTGTGGAAGAAGAACAGGGGGAG	CGTTGGCCAGGCACTTGCG
*Nkcc1*	GCCCAACGTGAGCTTCCAGAACG	CCTCAAAAGGTTCCTTTTCCAGCTCATCGT
*Nkcc2*	AGGCCTGGGAATCAGGAGTGCTATG	CCGCTCACGCTGCCTGTGT
*Gria1*	TGTCTACATTTATGATGCTGACCGGGGC	TCGGGAGTCACTTGTCCTCCATTGC
*Gria2*	GCATACAGATAGGGGGGCTATTTCCAAGG	TGCAGTGTTGATAAGCCTCTGTCACTGTC
*Grin2a*	GCTGACAAGGATCCGACATCCACG	GATGGAAACTCTTTGGGGATGAGCTCTGT
*Grin2b*	GGTGAGGTGGTCATGAAGAGGGC	GGGTTCTGCACAGGTACGGAGTTG
*KA1*	GGAGGATGAGGCGGGGACC	GCATGCTCTTCGGGAGGCTTCAAAAC
*KA2*	GGATGGGAAATATGGAGCCCAGGATGAT	TCAGGGGAGAGAGGATTCAGGAAGGAG
*Pkca*	CCAACGACTCCACGGCGTCTC	TGCTTGTGAACATTCATGTCGCAGGTGT
*Pkce*	TGATCATCGATCTCTCGGGATCATCGGG	GCCCACCTCGTCAGGGGTTTC
*Pik3ca*	CTGAGATGGGAGCTGGGACTGC	GTGTCCACGTGTTAGACAGAACACTG
*Pik3cb*	GAGGTTATGAGTGTGCTTCCGCCCTAT	AGTCTTCGTGTTTCGTCTTCCAGTTCCTC
*Pik3cg*	GCTGCGGAGTTCTACCACCGATTG	CAGGTAGTCTGGGAGAGGTTTGGACG

## Data Availability

The data presented in this study are available on request from the corresponding author.
